# Minimally invasive closed-chest ultrasound-guided substance delivery into the pericardial space in mice

**DOI:** 10.1007/s00210-012-0815-2

**Published:** 2012-12-19

**Authors:** S. Laakmann, L. Fortmüller, I. Piccini, S. Grote-Wessels, W. Schmitz, G. Breves, P. Kirchhof, L. Fabritz

**Affiliations:** 1 Department of Cardiology and Angiology, University Hospital Münster, Münster, Germany; 2 Institut für Pharmakologie und Toxikologie, University Hospital Münster, Münster, Germany; 3 Physiologisches Institut, Stiftung Tierärztliche Hochschule Hannover, Hannover, Germany; 4 Centre for Cardiovascular Sciences, School of Clinical and Experimental Medicine, University of Birmingham, Birmingham, UK

**Keywords:** Pericardial injection, Transgenic mice, Murine echocardiography, Gene transfer

## Abstract

Organ-directed gene transfer remains an attractive method for both gaining a better understanding of heart disease and for cardiac therapy. However, virally mediated transfer of gene products into cardiac cells requires prolonged exposure of the myocardium to the viral substrate. Pericardial injection of viral vectors has been proposed and used with some success to achieve myocardial transfection and may be a suitable approach for transfection of atrial myocardium. Indeed, such an organ-specific method would be particularly useful to reverse phenotypes in young and adult genetically altered murine models of cardiac disease. We therefore sought to develop a minimally invasive technique for pericardial injection of substances in mice. Pericardial access in anaesthetised, spontaneously breathing mice was achieved using continuous high-resolution ultrasound guidance. We could demonstrate adequate delivery of injected substances into the murine pericardium. Atrial epicardial and myocardial cells were transfected in approximately one third of mice injected with enhanced green fluorescent protein-expressing adenovirus. Cellular expression rates within individual murine atria were limited to a maximum of 20 %; therefore, expression efficiency needs to be further improved. Minimally invasive, ultrasound-guided injection of viral material appears a technically challenging yet feasible method for selective transfection of atrial epi- and myocardium. This pericardial injection method may be useful in the evaluation of potential genetic interventions aimed at rescuing atrial phenotypes in transgenic mouse models.

**Electronic supplementary material:**

The online version of this article (doi:10.1007/s00210-012-0815-2) contains supplementary material, which is available to authorized users.

## Introduction

Organ-specific, transient modification of gene expression is an attractive method for the pathophysiological understanding of heart disease and carries hopes for the development of new treatment methods. In view of the ready availability of genetically altered models, genetically modified mice have become a predominating model organism for investigating molecular mechanisms of heart disease. An efficient but minimally invasive cardiac gene transfer technique for murine models would substantially advance the study of relevant signalling mechanisms associated with cardiomyopathy and their reversal in mice. Furthermore, it would enable the preclinical evaluation of cardiac gene therapy.

In order to transfect cardiac tissue in vivo, vectors carrying gene products are predominantly delivered via thoracotomy and are either directly injected into the inner cavum of the left ventricle or intramyocardially (pigs: Mack et al. 1998; rabbits: Safi et al. 1999; dogs: Von Harsdorf et al. 1993). Another technique hitherto mostly limited to larger animals but starting to be explored in mice is catheter-based intracoronary infusion (Xu et al. 2006; Kornowski et al. 2000; Li et al. 2011; Roth et al. 2004a, b).

Intramyocardial administration is limited by the circumscribed region of genetic expression surrounding the needle tract, the result of a lack of diffusion following delivery (Guzman et al. 1993; Gilgenkrantz et al. 1995; Li et al. 1995; Niwano et al. 2008). Multiple injections are usually not practicable on account of the relatively invasive nature of the aforementioned methods. Furthermore, multiple intramyocardial injections are often associated with local tissue damage (French et al. 1994; Magovern et al. 1996; Guzman et al. 1993).

Pericardial application of genetic vectors appears to be a promising approach that avoids the limitations associated with myocardial injections. Thus far, particularly in rodents but also in larger mammals, pericardial access has been gained mainly through thoracotomy, e.g. in pigs (thoracotomy: Lamping et al. 1997), dogs (thoracotomy, catheter: Lazarous et al. 1999), rats (thoracotomy: Grkovic et al. 2005; Aoki et al. 1997; Bott-Flügel et al. 2005) and mice (thoracotomy: Fromes et al. 1999; Roth et al. 2004a, b). Thoracotomy carries a 10–15 % risk of periprocedural mortality (Wu et al. 2003), and repeated treatment is impracticable.

Therefore, a method which avoids surgery and optimises genetic transfection in regions of the heart not easily accessible by direct injection, such as the atria or the right ventricle, would be preferable. Here, we report on the development of a method of echo-guided pericardial injection of viral vectors in mice.

## Materials and methods

In order to develop and validate pericardial, ultrasound-guided delivery of vectors, the following experiments were performed:

1. Pericardial injections with the diagnostic dye indocyanine green to verify in vivo delivery of substances to the pericardial space and the heart ex vivo.
2. Pericardial injections with recombinant adenovirus expressing enhanced green fluorescent protein (EGFP).

This method was used in wild-type (WT) mice and in murine models of atrial pathology to test whether precise injection is feasible in physiological and pathologically altered hearts.

### Animals

During the first episode of the study, experiments were performed in WT mice on a CD1 and FVB strain background. Morphological changes in transgenic hearts modify conditions for cardiac injections; therefore, sex- and age-matched pairs of WT mice and transgenic mice under control of the α-MHC promoter were also injected in all episodes of the protocol. The genotypes were identified by polymerase chain reaction with the use of genomic tail DNA as previously reported.

All experiments and animal care procedures were approved by the local animal care and use committee (64-M1.18/06).

### Echocardiographically guided injections

With a view to performing cardiac gene therapy by injecting recombinant adenovirus into the pericardial sac of transgenic mice, we developed a method of ultrasound-guided percutaneous injections. This technique was used in all experiments reported here.

**Fig. 1:**
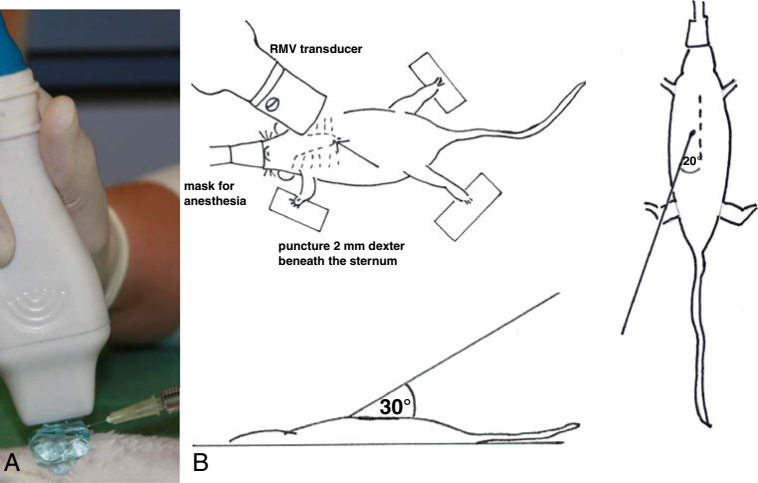
Method of pericardial injection. **a** Photographic illustration of transducer location and syringe during injection. **b** Scheme of experimental setup with anaesthetised mouse in supine position in top view and lateral perspective, needle entry 2 mm right beneath the edge of the sternum; transdiaphragmal direction towards the heart at ∼30° to the handling table surface

**Fig. 2:**
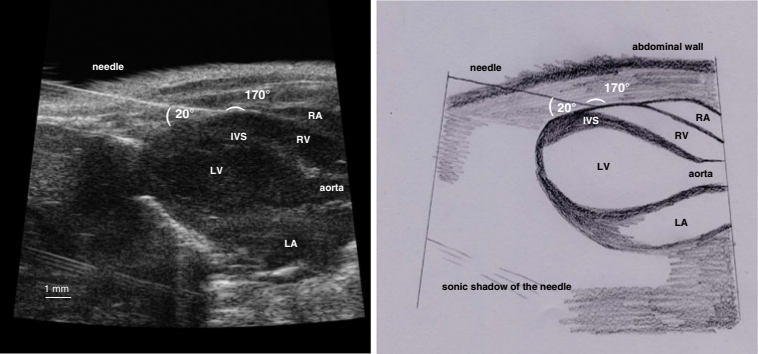
Echocardiographic visualisation of pericardial injection: modified echocardiographic LAV showing the position of the needle tip inside the pericardial sac for the pericardial injection in characteristic RV position (*left*) and scheme (*right*). *IVS* interventricular septum, *LA* left atrium, *LV* left ventricle, *RA* right atrium, *RV* right ventricle

Mice were anaesthetised with 2 % isoflurane (98 % oxygen) and hearts were visualised using a high-resolution Vevo 770 system and a 20- to 60-MHz real-time microvisualisation scanhead probe, respectively, a Vevo 2100 system and a 13- to 24-MHz MicroScan™Transducer (Visual Sonics, Toronto, Canada). Anaesthesia depth was closely monitored and the isoflurane dose adjusted accordingly. The chests of the animals were shaved and further cleaned by a chemical hair remover to minimise ultrasound attenuation and artefacts. Anaesthetised mice were fixed in a supine position by atraumatic clinical tape. Before positioning the ultrasound probe, centrifuged ultrasonic gel free of trapped air was applied to the exterior thoracic surface. Of the injection substance, 70 μl was loaded into a 1-ml syringe fitted with a sterile disposable 30-gauge needle. The needle was inserted 2 mm beneath the right part of the sternum through the body wall and directed in a transdiaphragmal direction towards the heart at ∼30° to the mouse table surface (Fig. 1). Visualising a modified long axis view (LAV) in B mode, the needle was gently advanced under ultrasound guidance until the needle tip was positioned in the pericardial sac near the right atrium. Correct positioning of the needle was verified by echocardiographic control and tactile vibration recognised by the person injecting (Fig. 2). Of the substance, 70 μl was administered and the needle removed quickly under echocardiographic control. Vigour of the heart and heart rate were monitored throughout and immediately after injection.

In total, 298 injections were performed: 150 hearts were harvested within 24 h post-injection, whilst the remaining 148 mice were monitored over the following 7 days after the injection to enable any complications to be identified.

The number of injections performed illustrates the development of an effective injection method which involved the following developmental steps:

1. Formation and improvement of the experimental setup including material and procedures
  In pretests, alternative mouse positions, e.g. the lateral position usually used during echocardiographic assessment, and different echocardiographic views were tested. The supine position adopted from pericardiocentesis in humans provided the easiest access and the least complication. After alternative mouse positioning, four of nine animals showed complications, e.g. discomfort (4) and post-interventional death (1), and indocyanine green was found within the thorax. We therefore decided to pursue the supine position even though alternative positioning may be feasible, if practised. Lowering heart rate to about 350 bpm during the procedure facilitated monitoring of the injection via echocardiography and positioning the needle and was easily achieved by sparing heating during anaesthesia.
  One hundred fifty-eight injections were performed using disposable syringes (B. Braun, Injekt®-F, 0.15-0.30 Euro/item), 97 with autoclaved Hamilton syringes (MicroliterTM, no. 710; ground glass tip, Luer Tip, needle not included, between 70.00 and 90.00 euro/item) and 43 with insulin syringes (B. Braun, Omnican 40, 0.10–0.24 euro/item). Light and cost-effective disposable syringes with an extra needle showed the highest resistance during injection, but resistance is unwanted during quick and localised handheld manipulation. Hamilton syringes were initially more smooth to inject with, but became less smooth-running after repeated use in our experiments (after approximately 20 injections). Insulin syringes (43) with a fixed needle turned out to be a good compromise, being easy to handle and reasonably priced. The learning curve shows no difference in success rate between the use of Hamilton syringes and disposable insulin syringes (Fig. 4).
2. Optimisation of injection substances including pretests with different volumes and supplements
  The diagnostic dye indocyanine green (70 µl, 11 mg/kg) was tolerated by the mice without any changes in general condition or behaviour. In contrast, injection of 50 µl or 15 mg/kg methylene blue was associated with death in three of three mice injected within hours after pericardial injection, possibly caused by a fatal methylene toxicity in connection with serotonin syndrome (Ng and Cameron 2010; Gillman 2010; Rowley et al. 2009).
  To facilitate penetration of the dye through the pericardial wall, we tested the use of collagenase and hyaluronidase, enzymes that degrade the extracellular matrix. During pretests with collagenase (20 µl, 4 mg/kg) alone or in combination with hyaluronidase (50 µl, 5805 U/kg), 7 of 11 mice died shortly after pericardial injections, and the other four were euthanised thereafter to avoid discomfort. Autopsy revealed in 7 of 11 cases a rupture of the diaphragm, three times with prolapse of the liver, and in four cases pulmonary haemorrhage. In contrast, hyaluronidase alone was well tolerated by the mice. We therefore concluded that this volume and concentration of collagenase and hyaluronidase used in combination was too aggressively digesting adjacent tissues.
  Finally, we tested different injecting volumes of indocyanine green (9× <50 μl, 76× 50 μl, 96× 60 μl, 80× 70 μl, 30× 80 μl, 7× 100 μl). Mice tolerated injecting volumes from 50 to 80 μl without any restrictions in general condition. Behaviour was unchanged and the fur well-groomed; activity did not decrease. Injection of 100 μl prolonged the recovery time.
3. Adaptation of experimental setup to genetically modified mice with altered hearts and injection conditions
  After 175 pericardial injections in WT mice, additional 108 injections in alpha MHC-expressing mice with enlarged atria were performed. The slight drop in the success rate after 180 injections (Fig. 4) might illustrate that adaptation to a different heart anatomy was necessary until previous results were reproducible.
4. Cardiac injections with recombinant adenovirus expressing EGFP to assess effectiveness of transfection
  After described pretests, we performed cardiac injections with recombinant adenovirus expressing EGFP underlying former described conditions. Mice were lying in a supine position and the hearts were visualised by echocardiography in a modified long axis view. Heart rate was lowered to 350 bpm. Hamilton syringes or disposable insulin syringes were used. Injecting volume was 70 μl containing hyaluronidase dissolved in a phosphate buffer (30 μl) with viral stock solution (40 μl).

### Pericardial application of indocyanine green

Indocyanine green (70 μl; Cardio Green, Indocyanine Green; Sigma-Aldrich Chemie GmbH, Steinheim, Germany) was injected into the pericardium using the injecting method described above. Within 24 h after injection, the hearts as well as bordering structures (lung, mediastinum and diaphragm) were examined for indocyanine green staining and removed under deep terminal anaesthesia. Tissues were also studied for scar tissue, necrosis and punctures. Abdominal organs were inspected.

### Pericardial application of recombinant adenovirus

The E1/E3-deleted replication-deficient adenovirus vector was constructed and propagated in HEK 293 cells according to standard protocols using the AdEasy system (Xu et al. 2006; He et al. 1998). In this system, a recombinant adenoviral plasmid is generated by cloning the gene of interest into the viral backbone using a minimal number of enzymatic manipulations. Transfection of this plasmid into the HEK 293 cell line facilitates viral propagation, producing a high titre of viral lysate containing the gene of interest tagged with EGFP. Visualisation of fluorescence facilitates monitoring of expression (He et al. 1998).

An adenoviral vector was chosen for this application due to its previous use as a temporary vector for gene transfer into the heart (Gilgenkrantz et al. 1995; Magovern et al. 1996) by methods including direct injection or coronary infusions (Guzman et al. 1993; French et al. 1994; Wasala 2011). The transduction solution injected contained hyaluronidase dissolved in a phosphate buffer (30 μl) with viral stock solution (40 μl). Injected hearts were harvested 4–10 days post-injection and examined for EGFP expression.

### Analysis of atrial EGFP expression

Atrial EGFP-expressing syncytial cells were counted using direct fluorescence microscopy. Hearts were embedded in paraffin and heart sections (5 μm in thickness) were cut either through the separated atria or along the longitudinal axis of the heart. Atrial expression rates were calculated using the total number of myocytes on the same section as a denominator.

### Electrocardiological experiments

Animals receiving adenovirus underwent electrophysiological evaluation before and after injection. In eight freely roaming mice, telemetric long-term Holter ECG recordings (EMKA Technologies, Paris, France) were performed following published methods (Fabritz et al. 2010; Kirchhof et al. 2006; Bett et al. 1994). Similarly, six-lead electrocardiograms (EMKA Technologies) were recorded and analysed every 24 h in nine mice for 4–10 days (Fabritz et al. 2004).

Heart rate (HR) and arrhythmia were assessed at baseline directly before and after injection during challenge with isoproterenol and by hot air jet stress (Froese et al. 2012; Wittköpper et al. 2010; Kirchhof et al. 2006; Bett et al. 1994).

### Echocardiographic evaluation

Mice injected with adenoviral vector underwent echocardiographic evaluation before and 8 days after injection. Cardiac morphology, size and function following standard protocols for anaesthetised mice (oxygenated isoflurane 1.5 % by inhalation, *n* = 5 per group, Vevo 770 system) were evaluated (Kirchhof et al. 2003, 2006, 2011, 2012; Fabritz et al. 2004, 2011). Echocardiography was also used to determine left atrial size and left atrial function in vivo (Blana et al. 2010).

### Statistical analysis

The data are presented as the mean ± SEM. Statistical differences were determined using Student’s *t* test and Fisher’s exact test. Two-sided *p* values <0.05 were considered significant.

## Results

### Peri-interventional survival

Two hundred ninety-eight cardiac injections were performed in 190 wild-type mice of different strains and in 108 alpha MHC-expressing mice with enlarged atria (mean age, 13 ± 1 weeks). Two hundred of the echo-guided injections were accomplished using indocyanine green, 39 with the recombinant adenovirus (mean age, 8 ± 1 weeks).

In general, mice recovered quickly after injections. However, 9 of 298 mice died during or immediately after injection, corresponding to a mortality rate of 3 %, mostly related to procedural complications: Four mice showed thrombotic material inside the thorax or abdomen as a sign of thoracic or abdominal lacerations, including one splenic rupture; one animal showed intraventricular necrosis, one showed pericardial tamponade, and one developed an air embolisation after unintended intraventricular injection.

Complications during early optimisation of the experimental setup, e.g. injection of methylene blue or application of a combination of collagenase and hyaluronidase, were not included in the peri-interventional survival statistics.

### Demonstration of substance delivery into the pericardial space

#### Analysis following indocyanine green injection

**Fig. 3:**
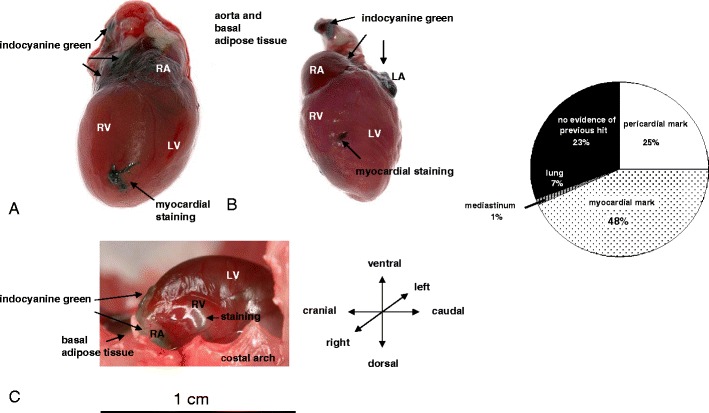
Detection of indocyanine green in myocardium (**a**, **b**) and pericardial sac (**a**–**c**) after echocardiography-guided injections. **c** The heart still inside the thorax in long axis view. *Right*, Circle diagram illustrating the distribution of needle marks after pericardial injections with indocyanine green

Upon autopsy, indocyanine green was still found in the pericardial space in 60 of 239 mice (25 %; Fig. 3). In the majority of mice, the myocardium was superficially treated in addition to the pericardial sac. Only in 8 % of mice was the pericardium treated without any evidence of superficial treatment of myocardium (19 of 239). At autopsy, indocyanine green was observed to stain myocardium in 48 % of mice (103 of 239). Superficial myocardial marks were observed. Myocardial administration was not deep and indocyanine green was not detected inside the lumen of the ventricle or atria. In 7 % of mice (17 of 239), staining of the lungs was detected (Fig. 3).

The diagnostic dye inocyanine green stained mainly the atria. Where the pericard was stained (in 60 of 239 % injections), 58 % was documented as atrial, 10 % in the pericard alongside the RV, 2 % alongside the LV, and in 30 %, indocyanine green was either spread all over the pericardium or a part not documented in detail. From the experiments with indocyanine green, we learnt that the atria were stained more intensively than the ventricles. Therefore, we concentrated our efforts on the atria regarding GFP expression.

**Fig. 4:**
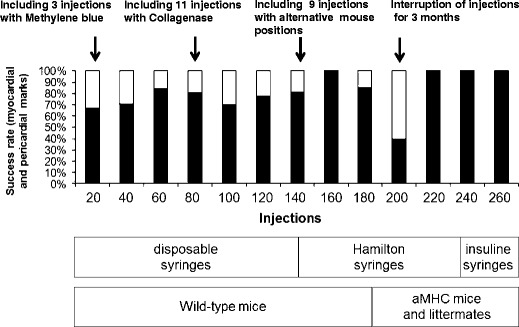
Learning curve for echocardiography-guided injections. Pitfalls and critical steps are labelled *above* and changes in injection conditions described *beneath learning curve bars*. Interruption of injections for nearly 3 months after injection; number 192 resulted in a drop in success rate

Injection success rate steadily improved: after 160 interventions, successful pericardial application/myocardial staining almost reached 100 %. Interruptions in practise for extended periods of time or change in substrate (e.g. different age group or genetically altered mice) may result in a drop in the success rate (Fig. 4).

#### Gene transfer efficiency after application of EGFP adenovirus

**Fig. 5:**
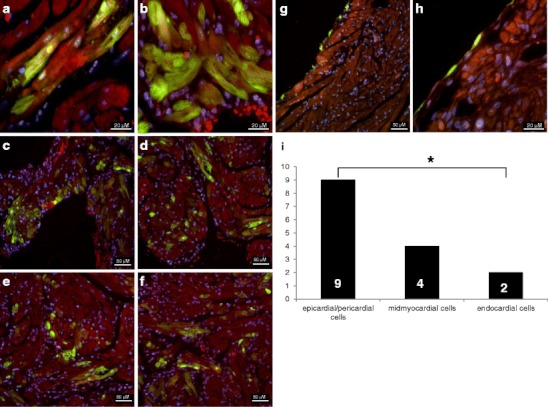
Atrial EGFP expression after pericardial injection: Transduction of midmyocardial cells (**a**, **b**), transfection of different cell layers (**c**–**f**) and visceral pericardial cells (**g**, **h**). Higher magnification (**h**) reveals that myocardial cells are not affected in this example. Nuclei were stained with DAPI DNA stain, appearing *blue*. **i** Distribution of GFP transfection in different cardial cell layers throughout the atria. Endocardial cells are significantly less transduced than epicardial or pericardial, with the trial midmyocardium showing intermediate expression

After establishing and optimising the technique for pericardial injection, we went on to assess the feasibility of transducing myocardial cells by injection of an EGFP-containing adenovirus (8–10 μl) into the pericardium. The fraction of EGFP-expressing cells in the atria varied from 8 to 20 % (Fig. 5).

At 6 weeks of age, the success rate of perimyocardial transfection was 37 % (10 of 27 hearts). Our analyses after GFP injection following the described injection method revealed GFP transduction of the right and left atrium in equal measure. In the 13 hearts of the investigated subgroup, transduction was found seven times in the LA and seven times in the RA.

To study expression differences within the atrial tissue, we counted the occurrence of transfections in a subgroup of samples at different localisations within the atrial tissues and compared the results using Fisher’s exact test. The major group of fluorescent cells was located pericardially or epicardially rather than endocardially (*p* < 0.05), with the atrial myocardium expressing an intermediate number of fluorescent cells (Fig. 5).

### Pericardial injection does not alter cardiac function

**Fig. 6:**
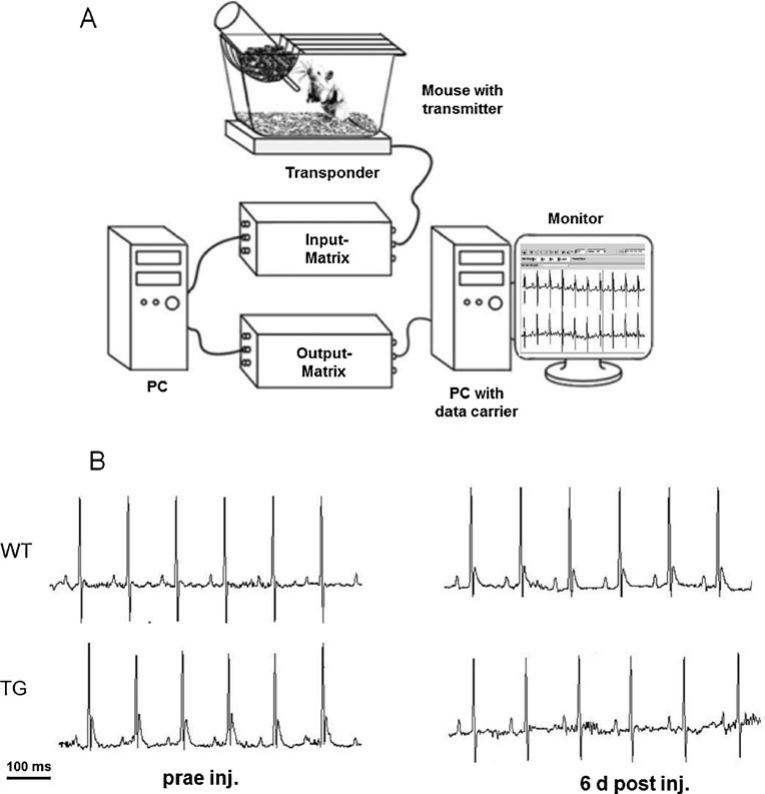
Telemetric investigation before and after pericardial injection. **a** Telemetric equipment. **b** Telemetric ECG recording from a freely moving WT mouse and a TG mouse during normal activity before (*left*) and after (*right*) echo-guided injection

Under freely roaming conditions (Fig. 6a, b), resting heart rate and maximal heart rate did not differ before and after pericardial injections (resting HR: *n* = 7, 3 WT, 4 transgenic (TG); WT = 487 ± 11 vs. 443 ± 12 bpm; TG = 488 ± 14 vs. 470 ± 14 bpm; maximal HR: WT = 592 ± 34 vs. 582 ± 23 bpm; TG = 701 ± 75 vs. 549 ± 22 bpm).

During air jet stress tests, maximal HR was comparable before and after injection (*n* = 7, 781 ± 14 vs. 816 ± 48 bpm), whilst the mean HR was lower after injection (*n* = 7, 664 ± 15 vs. 572 ± 28 bpm, *p* < 0.05). Cardiac function as assessed by serial transthoracic echocardiography before and after injection did not differ (Table 1).

**Table 1:** Cardiac dimensions in WT and TG mice as measured by high-resolution echocardiography before and 8 days after echo-guided cardiac injection

Parameters		Pre-injection		8 days post-injection		*p* values	
		WT	TG	WT	TG	Pre vs. post	
						WT	TG
Atria	Body weight (g)	27.1 ± 2.5	30.5 ± 1.5	27.1 ± 2.5	30.2 ± 1.8	0.996	0.919
	HR (bpm)	391 ± 20	379 ± 37	458 ± 22	417 ± 41	0.061	0.518
	LAs (mm)	1.26 ± 0.18	1.54 ± 0.26	1.26 ± 0.24	1.96 ± 0.20	0.996	0.249
	LA FS (%)	27 ± 5	25 ± 4	26 ± 2	22 ± 2	0.755	0.557
	MV E (cm/s)	87.1 ± 13.6	71.0 ± 11.9	91.4 ± 12.2	73.9 ± 1.8	0.821	0.824
	MV A (cm/s)	45.6 ± 5.2	46.3 ± 9.5	55.0 ± 1.5	50.7 ± 0.5	0.165	0.674
	IVSd (mm)	0.87 ± 0.03	0.80 ± 0.03	0.80 ± 0.02	0.77 ± 0.01	0.121	0.446
Ventricle	PWEDd (mm)	0.81 ± 0.02	0.81 ± 0.03	0.81 ± 0.02	0.91 ± 0.06	0.884	0.216
	LVEDd (mm)	3.96 ± 0.22	4.00 ± 0.11	3,92 ± 0.99	3.93 ± 0.12	0.850	0.673
	LVEDs (mm)	2.5 ± 0.09	2.70 ± 0.24	2.43 ± 0.19	2.74 ± 0.14	0.541	0.755
	FS (%)	32 ± 2	30 ± 2	35 ± 1	31 ± 5	0.284	0.846
	EF (%)	61 ± 2	58 ± 3	65 ± 2	59 ± 8	0.271	0.929
	CO (ml/min)	24 ± 2	14 ± 4	17 ± 2	12 ± 1	0.069	0.673
	LV Mass (mg)	124.4 ± 10.0	117.9 ± 2.2	115.4 ± 7.6	121.4 ± 2.5	0.484	0.331

*HR* heart rate, *LA* left atrial, *d* diastolic, *s* systolic, *FS* fractional shortening, *MV* mitral valve Doppler, *IVS* intraventricular septum, *PWEDd* posterior wall end diastolic diameter, *LVEDs* left ventricular end systolic diameter, *EF* ejection fraction, *CO* cardiac output

**Fig. 7:**
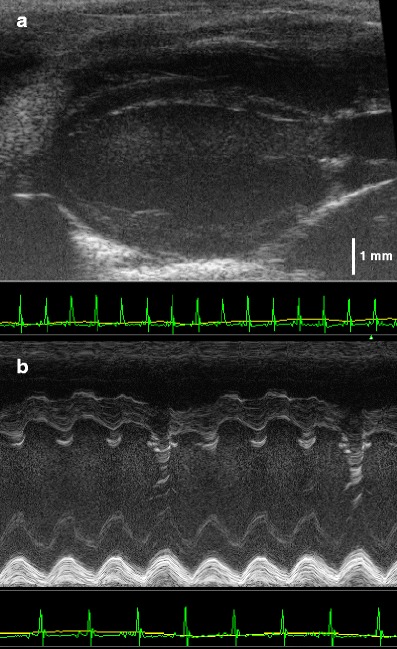
Echocardiographic parasternal long axis view two-dimensional (**a**) and motion (*M*) mode (**b**) of a WT mouse 8 days after pericardial injection. No evidence for pericardial effusion

Nevertheless, we monitored HR in the same cohort during daily six-lead electrocardiograms in sedation. HR was significantly increased in these mice if the pre- and post-injection hearts rate were compared for WT and TG mice together as one group from day 3 following pericardial injections of GFP virus solution to day 9 (*n* = 9: *n* = 4 WT, *n* = 5 TG, 399 ± 16 bpm pre-injection vs. 447 ± 24 bpm at 3 days, 450 ± 22 bpm at 4 days, 446 ± 21 bpm at 5 days, 472 ± 26 bpm at 6 days, 466 ± 17 bpm at 7 days, 463 ± 19 bpm at 8 days, 445 ± 27 bpm at 9 days, *p* < 0.05). Considering these results, the presence of some pericardial effusion induced by the pericardial injection is suggested, but we did not detect signs of pericardial effusion when performing echocardiographic assessments at day 8 after injection (Fig. 7).

Overall, there was no conclusive evidence of alteration of cardiac function.

## Discussion

Here, we report an ultrasound-guided technique for minimally invasive atrial-targeted substance and gene delivery in mice. Mice regained consciousness only minutes after minimally invasive injections, and procedures lasted only a few minutes. Rare mortality (3 %) was due to misplacement of the needle outside of the pericardial space, indicative of the technical skills required for successful injection.

Indocyanine green, the dye used during the introductory phase of the study, proved a reliable dye to track pericardial delivery. Mice tolerated the diagnostic dye without any changes in general condition or behaviour, and the dye was found in the pericardial space after euthanasia up to 24 h after injection. The dye was detected inside the myocardium as well as the pericardial cavity; however, it was also detected within the lung and mediastinal tissue in rare cases of needle misplacement.

The myocardium or pericardium was treated in 68 % of echo-guided injections with indocyanine green, which documents a satisfying success rate. In addition to being a short, well-tolerated procedure, careful post-procedural electrocardiographic and echocardiographic monitoring did not identify physiologically relevant changes in vital parameters or cardiac function. Furthermore, transduction of pericardial and myocardial tissue EGFP-expressing adenoviruses was achieved in 37 % of mice aged 6 weeks and in 27 % of all mice studied.

In this study, ultrasound-guided percutaneous injections into the pericardial cavity of juvenile and adult mouse hearts are described for the first time. This type of application immerses the heart in the applied substance and thereby exposes it to the vector for a prolonged time, thereby making parts of the heart that are difficult to access directly, e.g. the left atrium, accessible.

To date, direct injection into the myocardium or catheter-based intracoronary infusion in mice has not been easily achievable, and the resulting transgene expression is limited to the local region around the needle tract or the myocardium supplied by the selective coronary artery (Guzman et al. 1993; Kass-Eisler et al. 1993; Barr et al. 1994; Logeart et al. 2001).

Unfortunately, direct injection into the myocardium results in severe local tissue damage, which makes it an inadequate therapeutic approach (Guzman et al. 1993; Magovern et al. 1996). Myocardial injections are associated with significant injury, fibrosis and necrosis at the injection site, resulting in the accumulation of macrophages and neutrophils (Aoki et al. 1997).

By following the noninvasive method of ultrasound-guided pericardial injections as described here, the mouse heart maintains a prolonged contact time with the vector. Due to the observed epicardial–endocardial transfection gradient, the technique allows selective atrial transfection of epicardial and myocardial tissues (Springer et al. 2005).

Effective pericardial injections have been described in other species such as dogs (Lamping et al. 1997), rats (Aoki et al. 1997) as well as in mice (Fromes et al. 1999), but have always been combined with the invasive procedure of thoracotomy. Our strategy offers the potential for repeated interventions because of the well-tolerated, minimally invasive injection technique, thus enabling experimental designs with clinically relevant operating schedules, e.g. days or weeks, after a surgical procedure or regularly for the application of locally acting medication.

Zhang et al. (1999) were able to transfect the RV and atria by percutaneous subxiphoid injection into the pericardial cavity of 4- to 5-day-old mice, but long-term survival was compromised in this study: only mice with patchy staining of superficial cell layers survived more than 2 weeks. The survival rate increased after reducing the injection volumes, but this also reduced the level of gene expression. In neonatal mice, the pericardium is extensively attached to the sternum (Nakatani et al. 1988) and, therefore, easier to reach by injection than in adult animals. Furthermore in neonates, pericardial pores are absent (Nakatani et al. 1988), further prolonging exposure to the viral suspension.

Our study demonstrates that atrial-targeted gene transfer by percutaneous injection is possible in adult mice, mice lacking the advantages of neonatal anatomy for this procedure. In adult mice, validation of correct needle position by ultrasound is suggested; selecting an RV injecting position near to the right atrium has led to a satisfactory success rate in this study.

Pericardial injection leads to both left and right atrial EGFP expression in this study. Gene expression was not limited to the area around the needle tract, unlike in myocardial injections, but the atria, exposed to the viral suspension, were also affected. Atrial EGFP expression was mainly observed in pericardial cells, but also extended into the myocardium.

### Limitations

As with other methods of administration, the expression levels were still limited in this study. To achieve higher expression in atrial myocardial cells, further investigations such as increasing the dose of recombinant adenovirus or employing a different strategy to permeate the pericardial barrier are required. The combined use of hyaluronidase and collagenase was described (Fromes et al. 1999), but not well tolerated by the mice in our experimental setup.

### Outlook

The development of genetic vectors that are not only heart-specific but also able to target cardiac subregions may be more effective for atrium-specific gene transfer. To date, atrium-specific gene transfer has been reported in porcine hearts by direct application of adenoviral vectors to the epicardial surface (Kikuchi et al. 2005).

Adeno-associated viral vectors (AAV) with organ-targeted restricted transgene expression are promising (Fromes et al. 1999; Müller et al. 2007; Zincarelli et al. 2010). A combination of noninvasive pericardial injection and tissue-specific vectors could potentially make the described strategy more effective (Bish et al. 2011). Another approach for optimisation may be ultrasound-targeted destruction of microbubbles (Ghanem et al. 2009; Walton et al. 2011; Fujii et al. 2011) loaded with AAV (Bish et al. 2011).

Very recently, pericardial application of AAV virus to neonatal murine pericardium resulted in cardiac vector expression and phenotype rescue (Denegri et al. 2012), demonstrating potential impact on cardiovascular research and therapy.

The closed-chest minimally invasive pericardial injection technique described in this study may be helpful in evaluating the therapeutic potential of cardiac genetic modifications in murine models, especially when targeting the atria.

## Electronic supplementary material

Below is the link to the electronic supplementary material.

ESM 1 (AVI 584 kb)
